# The Rorschach Test Evaluation in Chronic Childhood Migraine: A Preliminary Multicenter Case–Control Study

**DOI:** 10.3389/fneur.2017.00680

**Published:** 2017-12-12

**Authors:** Maria Esposito, Antonietta Messina, Vincenzo Monda, Ilaria Bitetti, Filomena Salerno, Francesco Precenzano, Simone Pisano, Tiziana Salvati, Antonella Gritti, Rosa Marotta, Serena Marianna Lavano, Francesco Lavano, Agata Maltese, Lucia Parisi, Margherita Salerno, Gabriele Tripi, Beatrice Gallai, Michele Roccella, Domenico Bove, Maria Ruberto, Roberto Toraldo, Giovanni Messina, Marco Carotenuto

**Affiliations:** ^1^Center for Childhood Headache, Clinic of Child and Adolescent Neuropsychiatry, Department of Mental and Physical Health, and Preventive Medicine, Università degli Studi della Campania “Luigi Vanvitelli”, Naples, Italy; ^2^Department of Experimental Medicine, Section of Human Physiology and Unit of Dietetics and Sports Medicine, Università degli Studi della Campania “Luigi Vanvitelli”, Naples, Italy; ^3^Department of Child and Adolescent Neuropsychiatry, University of Salerno, Salerno, Italy; ^4^Faculty of Education Science, University Suor Orsola Benincasa of Naples, Naples, Italy; ^5^Department of Medical and Surgical Science, University Magna Graecia, Catanzaro, Italy; ^6^Department of Health Sciences, University “Magna Graecia”, Catanzaro, Italy; ^7^Unit of Child and Adolescent Neuropsychiatry, University of Perugia, Perugia, Italy; ^8^Child Neuropsychiatry, Department of Psychology and Pedagogical Sciences, University of Palermo, Palermo, Italy; ^9^Department PROSAMI, University of Palermo, Palermo, Italy; ^10^Childhood Psychiatric Service for Neurodevelopmentals Disorders, Chinon, France; ^11^Centro per la Diagnosi e Cura dei Disturbi dell’apprendimento e del Comportamento Associazione per la ricerca scientifica Fusis, Alvignano, Italy; ^12^Department of Medical-Surgical and Dental Specialties, Università degli Studi della Campania “Luigi Vanvitelli”, Naples, Italy; ^13^Department of Clinical and Experimental Medicine, University of Foggia, Foggia, Italy

**Keywords:** migraine without aura, Rorschach test, personality traits, children and adolescents, effect of general maladaptivity

## Abstract

**Object:**

About 1.2–3.2% of children at 7 years of age with increasing age up to 4–19% in adolescents are suffering from migraine without aura (MwA). The aim of the present study is investigating the personality style associated with children and adolescents affected by MwA, administrating the Rorschach test, and comparing with typical developing healthy controls (TD).

**Methods:**

137 patients (74 males), aged 7.3–17.4 years (mean age 11.4, SD 3.02 years), affected by MwA according to the IHs-3 criteria. The Rorschach variables were treated as numerical variables and statistically tested with *t*-Student’s analysis.

**Results:**

No statistical differences were found between the MwA and TD for age (*p* = 0.55), and gender (*p* = 0.804). From the comparison between the two samples, MwA group shows lower W responses (*p* < 0.001), good quality W responses (*p* < 0.001), high frequency of detailed responses (*p* < 0.001), the presence of even minor form of good quality responses (*p* < 0.001), increased presence of animals answers (A%) (*p* < 0.001), more frequent trivial answers (Ban%) (*p* < 0.001).

**Discussion:**

Rorschach interpretation pinpointed many interesting and, perhaps, peculiar aspects in our MwA population such as a trend predisposition for: analytical reasoning rather than synthetic, ease/practicality rather than creativity, oppositionality rather than external adaptation to the environment that may be interpreted as effect of general maladaptivity.

## Introduction

Migraine without aura (MwA) may be considered as relevant disabling primary headache characterized by high frequency of painful attacks with a prevalence ranging from 1.2 to 3.2% at 7 years of age, tending to increase with age up to 4–19% in adolescents (1).

Migraine without aura is frequent in pediatric age with a variety of comorbidities (2) that impact many day life aspects such as regulation and quality of sleep (3–10), mood regulation (2, 11), cognitive quality (12–16), motor coordination (5–10, 12–14), self-esteem (5–10), parenting styles (5–10).

In this light, MwA should be considered as disabling condition in pediatric age, particularly for the high risk of chronicity in adolescents and adults, despite of treatment (17, 18). In general, MwA is also considered an important cause for school absenteeism, poor quality of life, and social skills with peers, mainly during childhood and adolescence (19, 20). In the last decades, many studies reported the high prevalence of psychiatric comorbidity in children and adolescents affected by primary headache and MwA to be considered mandatory in the correct therapeutic and assessment management. Despite all, few studies about the chronological relationship between MwA and psychological stressors are reported. Mood alteration such as alertness (21, 22), emotional tension (22), depressive tendency (23), constant irritability, chronic fatigue (24) often tend to precede the migraine attacks. Migraine may be considered also related to anxious symptoms (25) and stress and mood changes could be related to MwA, although is not well known how MwA and mood may interact.

Many reports through last decades have identified the major triggers for migraine attacks (26, 27) in stressor factors. In fact, physiological response to stress tend to activate the hypothalamus–pituitary–adrenal and sympathetic nervous system, with subjective feeling of external or internal restlessness and worry (28). On the other hand, the autonomic/vegetative response intensity is related directly to frequency, duration, intensity of stress, and subjective state health traits (29). In this light, psychological stress may play a relevant role not only in prodromal migraine phases (30, 31) but also for painful attacks frequency (32, 33), and for symptoms maintaining, and for shifting from painful episodic to chronic symptoms (34).

Moreover, the role of putative personality peculiar traits in migraineurs is still discussed and debated, mainly considering that MwA patients tend to show an increase in neurotic and anxious personality traits (35, 36), also due to life events (37, 38).

Particularly referring to Rorschach test (RT) evaluation among children and adolescents with MwA, this topic is not completely new as research object because, in 1986, Guidetti et al. (38) examining 46 subjects of mean age, 10.4 highlighted no differences in children with migraine.

The hypothesis in the present Italian multicenter report was the identification of peculiar personality organization among children and adolescents affected by MwA evaluated on the projective RT respect of typical developing healthy controls (TD). The large number of recruited MwA children and adolescents and healthy control subjects can be considered the novelty and the strength of the present study.

## Materials and Methods

137 Caucasian patients (74 males and 63 females), between 7.3 and 17.4 years (mean age 11.4, SD 3.02 years), diagnosed by MwA according to the IHs-3 criteria were consecutively recruited in each pediatric headache center or neuropsychiatric clinic participating to the study, between January 2010 and December 2014.

200 TD Caucasian subjects (112 males and 88 females, between 7.1 and 17.3 years, mean age 11.6, SD 3.01 years) were randomly recruited from primary and secondary school in Sicily, Calabria, and Umbria Regions.

Exclusion criteria were neurological or psychiatric diagnosed illness and headaches different from MwA.

Parents of children and adolescents of both groups gave written consent and the study design and protocol was approved by Departmental ethics committees at the University of Palermo, Perugia and Catanzaro (EUDract 2010-000453-40).

### RT Coding and Evaluation

In order to investigate personality profile of children affected by MwA, the projective RT has been administered to MwA and TD subjects. RT is a personality projective evaluation based on subject’s interpretations of 10 standard inkblot tables, in order to measure emotional, cognitive, and integration functioning (39).

In our multicenter research study, we scored the RT evaluation according to criteria reported by Balottin et al. (39). The Passi Tognazzo method was used to code RT protocols, independently (40), by expert scorers, blinded to the subjects’ diagnoses.

As reported by Balottin et al. in 2009 (39), the numerical data obtained from the coding of the RT were replaced as numerical variables in order to be quantified and compared between MwA and TD groups. Mainly, the RT variables with normative data (R, W%, D%, F%, F+%, M, FM, FC, CF, C, sum of the color responses, sum of the shading responses, A%, H%, Ban%) (Table 1) were transformed into dichotomous variables (normal or pathological) and their frequency of distribution was compared among the groups (Mwa and healthy controls).

**Table 1:** Shows mean values of numerical variables among MwA and healthy controls.

	Migraine without aura (*N* = 137)	Controls (*N* = 200)	*F*	*p*
R	15.05 ± 7.01	14.6 ± 5.48	0.44	NS
W%	46.21 ± 25.28	67.9 ± 19.3	79.57	<0.001
W+%	59.8 ± 14.9	68.9 ± 12.1	38.02	<0.001
D%	48.61 ± 22.31	32.4 ± 20.9	46.29	<0.001
F%	41.54 ± 21.31	64.2 ± 10.6	166.26	<0.001
F+%	35.53 ± 22.83	60.2 ± 7.9	198.99	<0.001
R%1.t.	30.42 ± 7.54	31.9 ± 8.4	2.74	NS
F+%l.t.	16.35 ± 23.86	39.5 ± 27.1	65.29	<0.001
A%	55.1 ± 20.1	42.9 ± 13.6	44.18	<0.001
H%	19.85 ± 13.64	19.4 ± 10.2	0.12	NS
Anat%	1.89 ± 3.6	2.51 ± 4.63	1.74	NS
Ban%	29.15 ± 14.73	19.4 ± 9.9	52.83	<0.001
Orig%	2.74 ± 1.43	2.91 ± 1.85	0.82	NS

### Statistical Analysis

The Student’s *t*-test analysis was used to compare within the two groups (MwA and TD) RT variables (R, W%, W+%, D%, F%, F+%, R% l.t., F+% l.t. A%, H%, Anat%, Ban%, and Orig%). *p*-Values <0.05 were identified as statistically significant.

## Results

Migraine without aura and healthy controls showed no differences group for age (*p* = 0.55) and gender distribution (*p* = 0.804).

Mean values of numerical variables among MwA and healthy controls were compared according to Balottin et al. (39) (Table 1).

**Figure 1:**
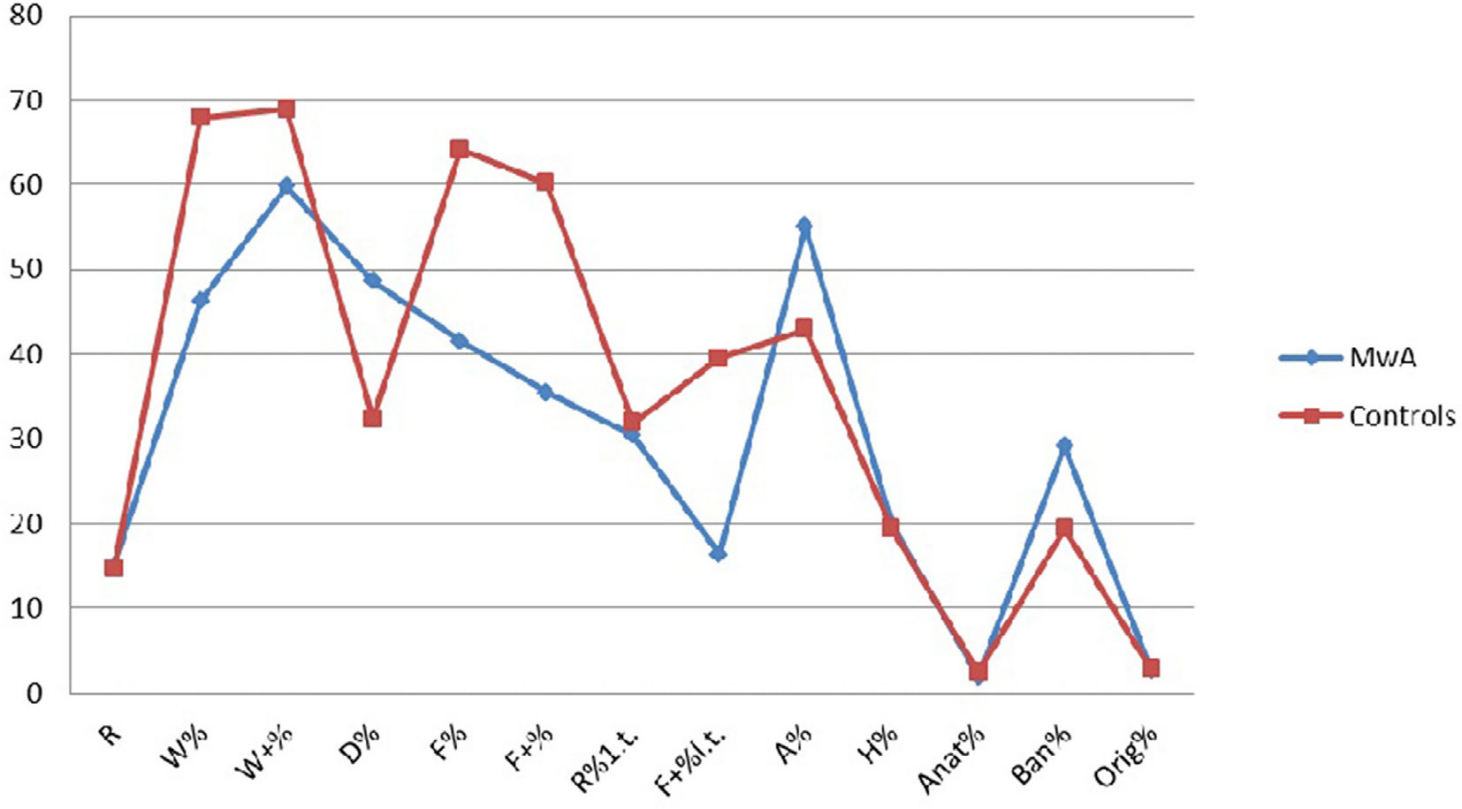
Summarizes the comparison among MwA group and healthy controls for RT evaluation.

From the comparison between the two samples, MwA group shows lower W responses (*p* < 0.001), good quality W responses (*p* < 0.001), high frequency among detailed responses (*p* < 0.001), the presence of even minor form of good quality responses (*p* < 0.001), increased presence of animals’ answers (A%) (*p* < 0.001), more frequent trivial answers (Ban%) (*p* < 0.001) (Figure 1).

## Discussion

Migraine is primary headache affecting about 7.8 million children in the European Union Countries (40). MwA is complex disease considered a continuum of events involving the CNS including painful sensation, cardiovascular changes, immunological changes (41–44).

Migraine without aura may be also interpreted as a maladaptive psychobiological disorder in which genetic predisposition interplays with a number of other factors, external or internal, including climatic, dietary, hormonal, psychological, and emotional factors (43).

In this light, MwA could be identified as the final effect of the loss of the ability to arrange homoeostatic changes against different multiple stressors (45) independently by age and gender.

Conversely, the Rorschach interpretation pinpointed many interesting and, perhaps, peculiar aspects in our MwA population such as a trend predisposition for: analytical reasoning rather than synthetic, ease/practicality rather than creativity, oppositionality rather than external adaptation to the environment that may be interpreted as effect of general maladaptivity. This aspect of environmental pathology (relational aspects after the 1 year of life; scholastic adaptation of child, his habits, and his/her family) are more evident than those regarding the primary mother–child relationship. According to the psychosomatic theory, we would have expected, instead, a prominent involvement of early diadic relationship.

Other findings such as stereotyped thought, conformism, hard reality testing, and psychological immaturity may be intended as effects of well-known specific cognitive ability.

On the other hand, the brain of migraineurs has been identified as different respect healthy controls (46–49), because dysmodulated (50–54). Morevoer, the gray matter decreasing has been identified among anterior cingulate cortex and insula regions (55), as direct effect of MwA attacks frequency or to the disease duration (56, 57), quite similar to depressed patients (58).

In resting state, functional connectivity studies among adolescents undergoing social stress has been identified as the connection between stress and medial prefrontal cortical regions, with cortisol activation related to anterior insula and medial prefrontal cortex regions also known as the “salience” network specifically involved in processing negative emotion such as anxiety and depression (59). This specific network is also involved in MwA complex pathogenesis (60, 61).

Partially at least, these neurobiological evidences could explain the complex interaction between personality traits and migraine, and why a large variability of neuropsychiatric comorbidities is frequently associated with migraine, including mood disorders (2, 11), anxiety (25), cognitive disability (12–16), and ADHD (62).

However, there are few studies that investigate personality and mood aspects of migraine, especially in childhood and adolescent age. Further studies may be important also to widen the therapeutic strategies. In this picture, chronic headaches should be treated with multidimensional approaches in order to support patients’ behavioral and cognitive strategies (63), as biofeedback and other biobehavioral therapies (64–75).

On the other hand, we have to take into account limitations of the present study: (1) the lack of follow-up study in order to verify the effects of psychotherapy and/or pharmacotherapy in these subjects; (2) the small number of recruited subjects.

In the light of all these considerations, further studies will be needed to better understand how personality aspects affect the symptoms of migraine, and how these aspects are related to each other and to the neurobiological bases of migraine in order to improve the management of migraine and of its associated disability.

## Ethics Statement

Parents of children and adolescents of both groups gave written consent and Departmental ethics committees at the University of Palermo, Perugia and Catanzaro approved the study protocol (EUDract 2010-000453-40).

## Author Contributions

ME, AM, VM, IB, FS, FP, MR, BG, RT: conceived the study, participated in its design. SP, TS, AG, RM, SML, FL, AgMa contributed to the conception and design. ME, AM, LP, MS, GT, MC wrote manuscript. LP, MS, BG, MiRo, GT, DB, drafted the article and revised it critically for important intellectual content. GM and MC: final approval of the version to be published. All authors read and approved the final manuscript.

## Conflict of Interest Statement

The authors declare that the research was conducted in the absence of any commercial or financial relationships that could be construed as a potential conflict of interest.
